# Refractory Ascites After Liver Transplantation Treated With Splenic Artery Embolization: A Case Report and Literature Review

**DOI:** 10.7759/cureus.43910

**Published:** 2023-08-22

**Authors:** Mauro Duvan Mendoza Quevedo, María Catalina Vaca-Espinosa, Juan Ignacio Marín Zuluaga, Brenda Carolina Amell Baron, Angie Karina Sierra Vargas

**Affiliations:** 1 Internal Medicine, University of the North, Barranquilla, COL; 2 General Medicine, University of Sabana, Bogotá, COL; 3 Hepatology, Pablo Tobon Uribe Hospital, Medellin, COL; 4 General Medicine, University of the North, Barranquilla, COL

**Keywords:** post-transplant ascites, liver transplantation, refractory ascites, splenic artery embolization, splenic artery steal syndrome

## Abstract

After orthotopic liver transplantation, several complications can arise, such as arterial or venous thrombosis, stenosis, biliary leakage, ischemia, or ascites. Although refractory ascites are not a common complication, they can have a significant impact on patients' prognosis and quality of life. This condition can be caused by multiple mechanisms, both intrahepatic and extrahepatic, and one of them is the splenic artery steal syndrome. In this article, we present the case of a patient with advanced cirrhosis who developed refractory ascites following liver transplantation. Despite management with diuretics and paracentesis, the ascites did not respond to conventional treatment. The diagnosis of splenic artery steal syndrome was confirmed through angiography, and subsequent embolization of the splenic artery resulted in symptom resolution.

## Introduction

Persistent ascites after liver transplantation are defined by the continued presence of ascitic fluid accumulation four weeks after undergoing orthotopic liver transplantation [1,2]. This condition occurs in around 3.5%-7% of patients who have undergone successful transplantation [3]. The reasons behind post-transplant ascites can be classified into two main groups: those external to the liver and those within the liver. Within the external group, they encompass blockage of the flow outside the liver due to stenosis in cava anastomosis, alongside chronic conditions of other organs like heart failure and chronic kidney disease. Also, bacterial or fungal infections have the potential to cause this condition. Regarding the liver-related factors, they include changes in blood flow within the liver due to portal vein thrombosis, ongoing portal hypertension, extended cold ischemia duration, graft rejection arising from reduced compliance of the hepatic vasculature, and the splenic artery steal syndrome [1,2]. 

The splenic artery steal syndrome was initially outlined by Langer et al. in 1990, highlighting it as an arterial "steal" phenomenon where a portion of blood from the hepatic artery's circulation diverts into the splenic artery stemming from the same source [4]. This condition has the potential to trigger early disruptions in liver function, bile duct damage due to inadequate blood supply, or even severe outcomes such as graft failure, which might necessitate retransplantation. Despite its potential consequences, this complication has received limited attention. According to available data, its occurrence has been documented, with varying incidence rates ranging from 0.6% to 10.1% [5]. In this study, we promptly identified a case of splenic artery steal syndrome following a liver transplant, with confirmation achieved through imaging studies and invasive hepatic hemodynamic assessments. Furthermore, common causes of ascites, both hepatic and extrahepatic, were ruled out. We believe that publishing this case is imperative due to its infrequent prevalence and potential for harm. This condition should be recognized by general practitioners and medical facilities that conduct orthotopic liver transplantation, with the objective of ensuring its prompt recognition and treatment. Moreover, recognizing post-transplant ascites as a condition with specific etiologies, such as splenic artery steal syndrome, holds paramount importance.

## Case presentation

We present the case of a 33-year-old woman with a history of autoimmune hepatitis-related cirrhosis and complications of portal hypertension, including ascites and esophageal varices. Due to advanced cirrhosis with a model for end-stage liver disease (MELD) score of 26, she underwent orthotopic liver transplantation (OLT) from a deceased donor. Right after the surgical procedure, the patient had an initial graft dysfunction requiring comprehensive intensive care support. This involved the administration of intravenous fluids, blood product transfusions, and high-dose corticosteroid immunosuppression. After a few weeks in the ICU, her condition improved in a remarkable way. From the first week post-transplantation, she developed ascites that persisted for more than four weeks, despite treatment with diuretics, requiring multiple large-volume paracentesis. The analysis of ascitic fluid revealed a yellow-colored liquid with a total protein concentration of 1.6 g/dL, an ascitic fluid albumin level of 0.9 g/dl, lactate dehydrogenase (LDH) measuring 170 U/L, and a white blood cell count of 37, with only 15% being polymorphonuclear cells. The serum albumin concentration was 2.5 g/dl, resulting in a serum ascitic albumin gradient (SAAG) of 1.6 g/dl. Hepatic function tests carried out in the fourth week post-transplantation indicated ALT at 10 IU/L, AST at 20 IU/L, a total bilirubin of 2.04 mg/dL, a direct bilirubin of 0.93 mg/dL, alkaline phosphatase (ALP) measuring 144 units/L, a prothrombin time of 13.2 seconds, and an international normalized ratio (INR) of 1.16. The administered immunosuppressive therapy included tacrolimus, prednisolone, and azathioprine. During her hospital stay, she also developed spontaneous bacterial peritonitis caused by ESBL-producing E. coli, which was treated with carbapenems.

**Figure 1:**
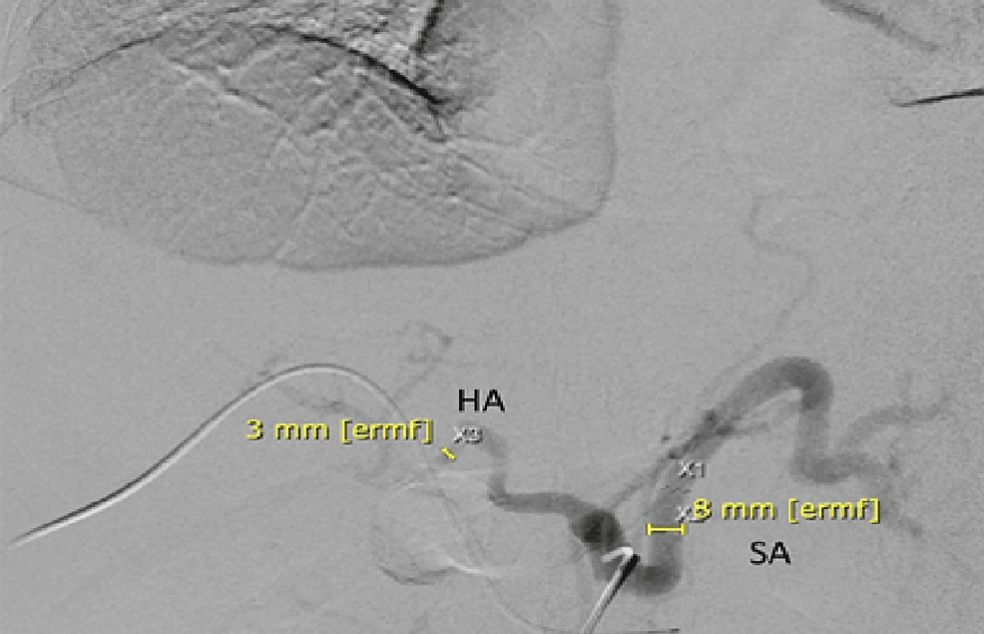
Splenic angiography revealed that the hepatic artery (HA) exhibited a reduced caliber of 3 mm and sluggish flow in comparison to the splenic artery (SA) 8 mm.

**Figure 2:**
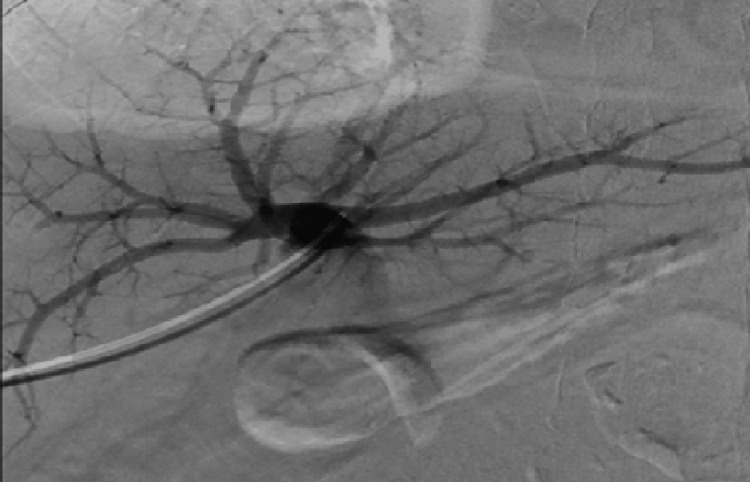
Hepatic portography revealed that the anastomosis was patent.

**Figure 3:**
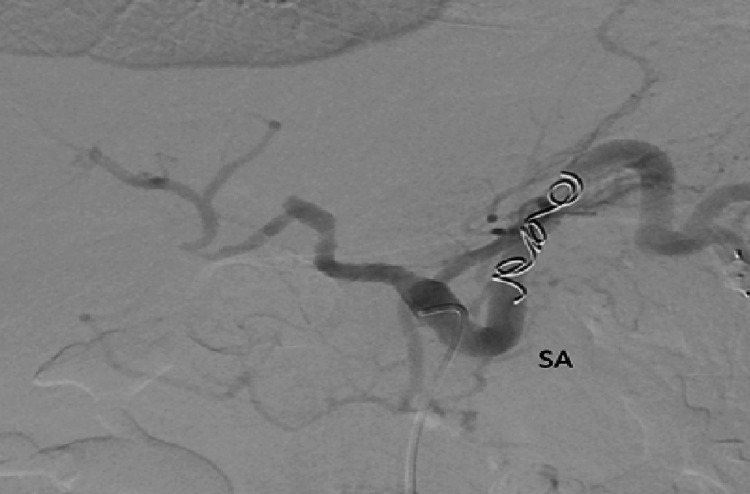
A successful embolization of the splenic artery (SA) was performed.

**Figure 4:**
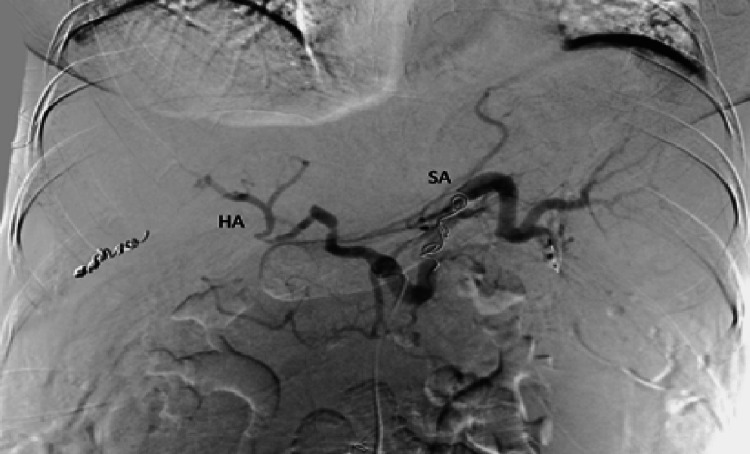
Splenic embolization with a coil in the middle portion of the splenic artery (SA).

A liver biopsy revealed areas of ischemic hepatic necrosis, mixed steatosis, and moderate inflammatory infiltrates in the portal spaces. Based on these findings, the cause of refractory ascites was attributed to post-transplant splenic artery steal syndrome. Successful embolization of the splenic artery was performed (Figures 3, 4). Following the procedure, the patient experienced resolution of ascites, allowing for the discontinuation of diuretics without the need for further paracentesis. Initially, an abdominal ultrasound was conducted, revealing the presence of ascites. The hepatic graft appeared normal in size with regular contours, and both arterial and venous patency of the graft were observed. Subsequently, a Doppler study was performed, demonstrating a portal vein of normal caliber measuring 10 mm in diameter with antegrade flow. The hepatic artery showed patency, characterized by peak systolic velocities of 181 cm/s at the hilum and a resistive index (RI) of 0.91. The hepatic veins exhibited biphasic flow and maintained patency. Following these assessments, an abdominal MRI was conducted, revealing adequate vascular patency and anastomotic function, with a significant amount of ascites associated with splenomegaly and splenorenal shunt and a liver/spleen ratio of 0.6. The splenic artery was found to have a diameter of >8 mm compared to the 3.7 mm diameter of the hepatic artery. Despite improvement in liver function tests, the persistence of ascites prompted an echocardiogram, which showed no abnormalities in cardiac function. Therefore, a transjugular liver biopsy and measurement of the hepatic venous pressure gradient were conducted, revealing evidence of a patent 12 mm anastomosis, with pressures in the splenic vein and main portal vein measuring 18 mmHg and 14 mmHg, respectively, a gradient of 4 mmHg. Additionally, celiac angiography was performed, revealing a reduced caliber (3 mm) and sluggish flow in the hepatic artery, in contrast to the splenic artery (8 mm) (Figures 1, 2).

A liver biopsy revealed areas of ischemic hepatic necrosis, mixed steatosis, and moderate inflammatory infiltrates in the portal spaces. Based on these findings, the cause of refractory ascites was attributed to post-transplant splenic artery steal syndrome. A successful coil embolization of the splenic artery was performed, leading to a noticeable reduction in splenic blood flow and improved hepatic perfusion extending to the peripheral areas (Figures 3, 4). A post-procedural Doppler assessment demonstrated sustained patency of the hepatic artery, characterized by a low-resistance flow pattern. The systolic velocity of the hepatic artery at the hilum was recorded as 94.9 cm/s, with a hepatic artery resistive index (RI) of 0.55. Furthermore, the portal vein was confirmed to maintain its patency. Following the procedure, the patient experienced resolution of ascites, allowing for the discontinuation of diuretics without the need for further paracentesis.

## Discussion

Refractory ascites after orthotopic liver transplantation is an infrequent complication, but it has a significant impact on the prognosis and quality of life of patients. It occurs in approximately 5% of patients undergoing orthotopic liver transplantation [2,6]. A significant increase in mortality has been observed in the first-year post-transplantation among patients with this complication [6]. The main causes of post-transplant ascites can be classified into hepatic and extrahepatic causes [1] (Table 1).

**Table 1:** Causes of ascites after liver transplantation.

Hepatic causes	Extra-hepatic causes
Disturbances in hepatic inflow	Heart failure
Disturbances in hepatic outflow	Chronic kidney disease
Hepatitis C recurrence	Infections (bacterial or fungal peritonitis)
Transplant rejection	
Prolonged cold ischemia time	

In the sequential evaluation of a patient with post-transplant ascites, it is important to start with a diagnostic paracentesis to rule out infectious causes and determine the characteristics of the ascitic fluid, typically obtaining a serum-ascites albumin gradient equal to or greater than 1.1 g/dL. In the clinical case, the analysis of the ascitic fluid revealed a yellow-colored liquid with an ascitic fluid albumin level of 0.9 g/dl, a serum ascitic albumin gradient (SAAG) of 1.6, and a white blood cell count of 37, with only 15% being polymorphonuclear cells. Then it is necessary to perform an abdominal ultrasound and doppler to exclude other causes of ascites; if the cause is not clear yet, a dynamic imaging such as abdominal CT or/and abdominal MRI study should also be performed to assess hepatic vascular permeability, and an evaluation of cardiac function through echocardiography to explore extrahepatic causes. If the underlying cause remains elusive, it is advisable to consider a transjugular liver biopsy, venography, and measurement of pressure gradients. Furthermore, in cases where there is suspicion of splenic artery steal syndrome, it is prudent to contemplate additional investigations, such as celiac angiography [1,7].

In the patient’s case, splenic artery steal syndrome was suspected as the cause of refractory post-transplant ascites. This suspicion arises primarily after ruling out other potential causes and due to the imaging findings of increased resistance in the hepatic artery, coupled with a reduced diameter of the hepatic artery as compared to the splenic artery. Splenic artery steal syndrome involves a selective diversion of blood flow from the liver allograft to an enlarged spleen through the splenic artery [3]. This entity causes hepatic hypoperfusion, leading to graft dysfunction and, in some cases, the development of ascites [8]. Although the pathophysiology is not clearly defined, two theories have been proposed to explain this phenomenon. The first theory suggests a diversion of arterial flow towards the splenic artery, resulting in decreased blood flow in the hepatic artery, thus producing an "arterial steal" phenomenon. The other theory is based on the generation of portal hyperperfusion, which leads to decreased flow in the hepatic artery due to a drop in adenosine concentrations in the portal triad [7].

The main pre-transplant predictive factors described for the splenic artery steal syndrome include splenomegaly with a volume >830 cm³ and undersized grafts (small-for-size syndrome). Doppler findings lack specificity, with the most commonly reported being an elevated arterial resistance index in the hepatic artery (RI: >0.80) [7]. It is noteworthy that this particular observation was evident in the presented patient case.

Angiography is the gold standard for diagnosis, revealing slowed flow in the hepatic artery in the absence of significant anatomical alterations such as stenosis, thrombosis, or vascular kinks. For the diagnosis of splenic artery steal syndrome, measurements of the splenic artery diameter > 4 mm and/or a splenic artery-to-hepatic artery diameter ratio > 1.5 have been utilized [7]. A spleen-to-liver volume index > 0.5 suggests selective diversion of flow and serves as a predictor for interventional treatment [9]. Regrettably, at present, there are no well-defined and validated objective criteria for diagnosing splenic artery steal syndrome. As seen in the previously presented case, characteristic dynamic angiographic observations frequently demonstrate rapid perfusion within the splenic while conversely revealing delayed or reduced perfusion within the hepatic artery. Moreover, the early enhancement of contrast within the portal venous system stands as a significant indicator of compromised hepatic arterial blood flow [10].

Embolization of the splenic artery has been shown to be a safe and effective procedure for the treatment of post-transplant ascites. Its mechanism is based on reducing portal hyperperfusion, which is the cause of hepatic arterial hypoperfusion, thus increasing hepatic arterial flow and consequently resolving complications such as refractory post-transplant ascites and hepatic hydrothorax. The main complications described include post-embolization syndrome, splenic abscess, splenic infarction, and splenic rupture [11]. Other surgical approaches encompass splenic artery banding and splenectomy, typically reserved for cases unresponsive to interventional treatments. Although surgical banding has been used to address SAS, it amplifies morbidity, particularly in high-risk surgical candidates. Splenectomy proves effective for confirmed SAS patients, yet post-operative sepsis and potential portal vein thrombosis serve as notable complications. Present guidelines advocate splenectomy primarily when coupled with accompanying conditions, such as splenic artery aneurysms [12].

## Conclusions

As orthotopic liver transplantation is part of the treatment for patients with chronic liver disease and its complications, it is necessary to consider its possible complications, including post-transplant refractory ascites. The approach to treating this complication should focus on identifying and correcting its underlying cause. Splenic artery steal syndrome represents a rare yet potentially manageable complication of post-transplant ascites. Hence, it is imperative to comprehend the diagnostic procedures and potential treatment modalities associated with this syndrome. In the presented clinical case, the patient was diagnosed with splenic artery steal syndrome after performing a celiac angiography, and it was successfully treated with splenic artery embolization, resulting in the resolution of symptoms. Although significant advances have been made in liver transplantation, further research is still needed to investigate the etiology and treatment of post-procedural complications. At present, there is a need for further studies aimed at validating predictive factors and establishing objective diagnostic criteria for patients who are suspected of having splenic artery steal syndrome after undergoing hepatic transplantation.
